# Lessons from the History of Quarantine, from Plague to Influenza A

**DOI:** 10.3201/eid1902.120312

**Published:** 2013-02

**Authors:** Eugenia Tognotti

**Affiliations:** Author affiliation: University of Sassari, Sassari, Sardinia, Italy

**Keywords:** infectious diseases, epidemics, public health measures, quarantine, isolation, maritime quarantine, sanitary cordons, lazarettos, public health emergencies, political issues, economic issues, social issues, ethical issues, viruses, bacteria, discrimination, stigmatization, prejudice, border controls, communicable diseases, influenza, cholera, Black Death, SARS, severe acute respiratory syndrome

## Abstract

In the new millennium, the centuries-old strategy of quarantine is becoming a powerful component of the public health response to emerging and reemerging infectious diseases. During the 2003 pandemic of severe acute respiratory syndrome, the use of quarantine, border controls, contact tracing, and surveillance proved effective in containing the global threat in just over 3 months. For centuries, these practices have been the cornerstone of organized responses to infectious disease outbreaks. However, the use of quarantine and other measures for controlling epidemic diseases has always been controversial because such strategies raise political, ethical, and socioeconomic issues and require a careful balance between public interest and individual rights. In a globalized world that is becoming ever more vulnerable to communicable diseases, a historical perspective can help clarify the use and implications of a still-valid public health strategy.

## Abstract 2

The complex and controversial history of this centuries-old public health strategy offers guidance for its future use.

The risk for deadly infectious diseases with pandemic potential (e.g., severe acute respiratory syndrome [SARS]) is increasing worldwide, as is the risk for resurgence of long-standing infectious diseases (e.g., tuberculosis) and for acts of biological terrorism. To lessen the risk from these new and resurging threats to public health, authorities are again using quarantine as a strategy for limiting the spread of communicable diseases (*1*). The history of quarantine—not in its narrower sense, but in the larger sense of restraining the movement of persons or goods on land or sea because of a contagious disease—has not been given much attention by historians of public health. Yet, a historical perspective of quarantine can contribute to a better understanding of its applications and can help trace the long roots of stigma and prejudice from the time of the Black Death and early outbreaks of cholera to the 1918 influenza pandemic (*2*) and to the first influenza pandemic of the twenty-first century, the 2009 influenza A(H1N1)pdm09 outbreak (*3*).

Quarantine (from the Italian “quaranta,” meaning 40) was adopted as an obligatory means of separating persons, animals, and goods that may have been exposed to a contagious disease. Since the fourteenth century, quarantine has been the cornerstone of a coordinated disease-control strategy, including isolation, sanitary cordons, bills of health issued to ships, fumigation, disinfection, and regulation of groups of persons who were believed to be responsible for spreading the infection (*4*,*5*).

## Plague

Organized institutional responses to disease control began during the plague epidemic of 1347–1352 (*6*). The plague was initially spread by sailors, rats, and cargo arriving in Sicily from the eastern Mediterranean (*6*,*7*); it quickly spread throughout Italy, decimating the populations of powerful city-states like Florence, Venice, and Genoa (*8*). The pestilence then moved from ports in Italy to ports in France and Spain (*9*). From northeastern Italy, the plague crossed the Alps and affected populations in Austria and central Europe. Toward the end of the fourteenth century, the epidemic had abated but not disappeared; outbreaks of pneumonic and septicemic plague occurred in different cities during the next 350 years (*8*).

Medicine was impotent against plague (*8*); the only way to escape infection was to avoid contact with infected persons and contaminated objects. Thus, some city-states prevented strangers from entering their cities, particularly, merchants (*10*) and minority groups, such as Jews and persons with leprosy. A sanitary cordon—not to be broken on pain of death—was imposed by armed guards along transit routes and at access points to cities. Implementation of these measures required rapid, firm action by authorities, including prompt mobilization of repressive police forces. A rigid separation between healthy and infected persons was initially accomplished through the use of makeshift camps (*10*). 

Quarantine was first introduced in 1377 in Dubrovnik on Croatia’s Dalmatian Coast (*11*), and the first permanent plague hospital (lazaretto) was opened by the Republic of Venice in 1423 on the small island of Santa Maria di Nazareth. The lazaretto was commonly referred to as Nazarethum or Lazarethum because of the resemblance of the word lazaretto to the biblical name Lazarus (*12*). In 1467, Genoa adopted the Venetian system, and in 1476 in Marseille, France, a hospital for persons with leprosy was converted into a lazaretto. Lazarettos were located far enough away from centers of habitation to restrict the spread of disease but close enough to transport the sick. Where possible, lazarettos were located so that a natural barrier, such as the sea or a river, separated them from the city; when natural barriers were not available, separation was achieved by encircling the lazaretto with a moat or ditch. In ports, lazarettos consisted of buildings used to isolate ship passengers and crew who had or were suspected of having plague. Merchandise from ships was unloaded to designated buildings. Procedures for so-called “purgation” of the various products were prescribed minutely; wool, yarn, cloth, leather, wigs, and blankets were considered the products most likely to transmit disease. Treatment of the goods consisted of continuous ventilation; wax and sponge were immersed in running water for 48 hours.

It is not known why 40 days was chosen as the length of isolation time needed to avoid contamination, but it may have derived from Hippocrates theories regarding acute illnesses. Another theory is that the number of days was connected to the Pythagorean theory of numbers. The number 4 had particular significance. Forty days was the period of the biblical travail of Jesus in the desert. Forty days were believed to represent the time necessary for dissipating the pestilential miasma from bodies and goods through the system of isolation, fumigation, and disinfection. In the centuries that followed, the system of isolation was improved (*13*–*15*). 

In connection with the Levantine trade, the next step taken to reduce the spread of disease was to establish bills of health that detailed the sanitary status of a ship’s port of origin (*14*). After notification of a fresh outbreak of plague along the eastern Mediterranean Sea, port cities to the west were closed to ships arriving from plague-infected areas (*15*). The first city to perfect a system of maritime cordons was Venice, which because of its particular geographic configuration and its prominence as a commercial center, was dangerously exposed (*12*,*15*,*16*). The arrival of boats suspected of carrying plague was signaled with a flag that would be seen by lookouts on the church tower of San Marco. The captain was taken in a lifeboat to the health magistrate’s office and was kept in an enclosure where he spoke through a window; thus, conversation took place at a safe distance. This precaution was based on a mistaken hypothesis (i.e., that “pestilential air” transmitted all communicable diseases), but the precaution did prevent direct person-to-person transmission through inhalation of contaminated aerosolized droplets. The captain had to show proof of the health of the sailors and passengers and provide information on the origin of merchandise on board. If there was suspicion of disease on the ship, the captain was ordered to proceed to the quarantine station, where passengers and crew were isolated and the vessel was thoroughly fumigated and retained for 40 days (*13*,*17*). This system, which was used by Italian cities, was later adopted by other European countries.

The first English quarantine regulations, drawn up in 1663, provided for the confinement (in the Thames estuary) of ships with suspected plague-infected passengers or crew. In 1683 in Marseille, new laws required that all persons suspected of having plague be quarantined and disinfected. In ports in North America, quarantine was introduced during the same decade that attempts were being made to control yellow fever, which first appeared in New York and Boston in 1688 and 1691, respectively (*18*). In some colonies, the fear of smallpox outbreaks, which coincided with the arrival of ships, induced health authorities to order mandatory home isolation of persons with smallpox (*19*), even though another controversial strategy, inoculation, was being used to protect against the disease. In the United States, quarantine legislation, which until 1796 was the responsibility of states, was implemented in port cities threatened by yellow fever from the West Indies (*18*). In 1720, quarantine measures were prescribed during an epidemic of plague that broke out in Marseille and ravaged the Mediterranean seaboard of France and caused great apprehension in England. In England, the Quarantine Act of 1710 was renewed in 1721 and 1733 and again in 1743 during the disastrous epidemic at Messina, Sicily (*19*). A system of active surveillance was established in the major Levantine cities. The network, formed by consuls of various countries, connected the great Mediterranean ports of western Europe (*15*).

## Cholera

By the eighteenth century, the appearance of yellow fever in Mediterranean ports of France, Spain, and Italy forced governments to introduce rules involving the use of quarantine (*18*). But in the nineteenth century, another, even more frightening scourge, cholera, was approaching (*20*). Cholera emerged during a period of increasing globalization caused by technological changes in transportation, a drastic decrease in travel time by steamships and railways, and a rise in trade. Cholera, the “Asiatic disease,” reached Europe in 1830 and the United States in 1832, terrifying the populations (*21**–**24*). Despite progress regarding the cause and transmission of cholera, there was no effective medical response (*25*).

During the first wave of cholera outbreaks, the strategies adopted by health officials were essentially those that had been used against plague. New lazarettos were planned at western ports, and an extensive structure was established near Bordeaux, France (*26*). At European ports, ships were barred entry if they had “unclean licenses” (i.e., ships arriving from regions where cholera was present) (*27*). In cities, authorities adopted social interventions and the traditional health tools. For example, travelers who had contact with infected persons or who came from a place where cholera was present were quarantined, and sick persons were forced into lazarettos. In general, local authorities tried to keep marginalized members of the population away from the cities (*27*). In 1836 in Naples, health officials hindered the free movement of prostitutes and beggars, who were considered carriers of contagion and, thus, a danger to the healthy urban population (*27*,*28*). This response involved powers of intervention unknown during normal times, and the actions generated widespread fear and resentment.

In some countries, the suspension of personal liberty provided the opportunity—using special laws—to stop political opposition. However, the cultural and social context differed from that in previous centuries. For example, the increasing use of quarantine and isolation conflicted with the affirmation of citizens’ rights and growing sentiments of personal freedom fostered by the French Revolution of 1789. In England, liberal reformers contested both quarantine and compulsory vaccination against smallpox. Social and political tensions created an explosive mixture, culminating in popular rebellions and uprisings, a phenomenon that affected numerous European countries (*29*). In the Italian states, in which revolutionary groups had taken the cause of unification and republicanism (*27*), cholera epidemics provided a justification (i.e., the enforcement of sanitary measures) for increasing police power. 

By the middle of the nineteenth century, an increasing number of scientists and health administrators began to allege the impotence of sanitary cordons and maritime quarantine against cholera. These old measures depended on the idea that contagion was spread through the interpersonal transmission of germs or by contaminated clothing and objects (*30*). This theory justified the severity of measures used against cholera; after all, it had worked well against the plague. The length of quarantine (40 days) exceeded the incubation period for the plague bacillus, providing sufficient time for the death of the infected fleas needed to transmit the disease and of the biological agent, *Yersinia pestis*. However, quarantine was almost irrelevant as a primary method for preventing yellow fever or cholera. A rigid maritime cordon could only be effective in protecting small islands. During the terrifying cholera epidemic of 1835–1836, the island of Sardinia was the only Italian region to escape cholera, thanks to surveillance by armed men who had orders to prevent, by force, any ship that attempted to disembark persons or cargo on the coast (*27*).

**Figure 1:**
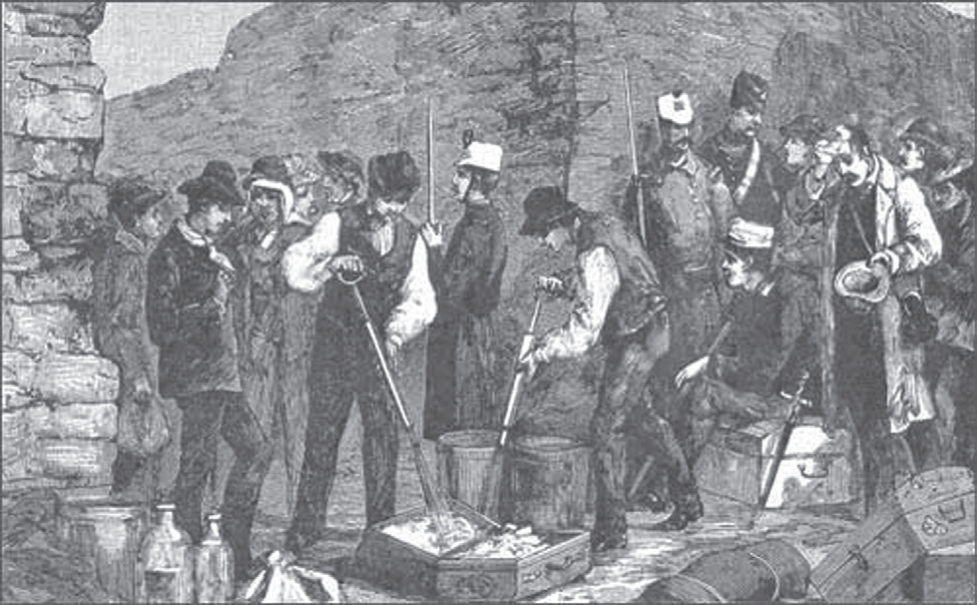
Disinfecting clothing. France–Italy border during the cholera epidemic of 1865–1866. (Photograph in the author's possession).

**Figure 2:**
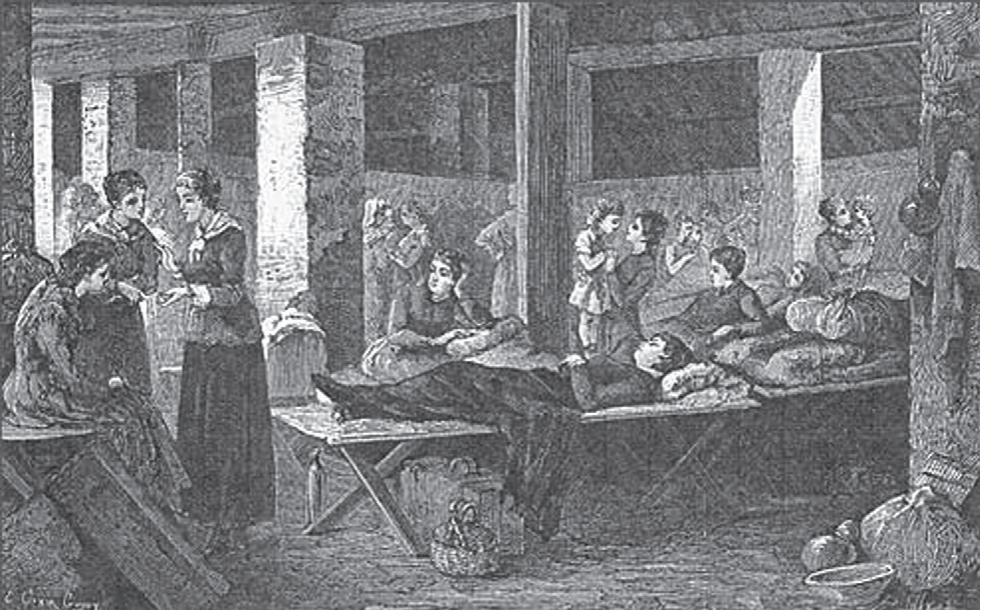
Quarantine. The female dormitory. France–Italy border during the cholera epidemic of 1865–1866. (Photograph in the author's possession).

**Figure 3:**
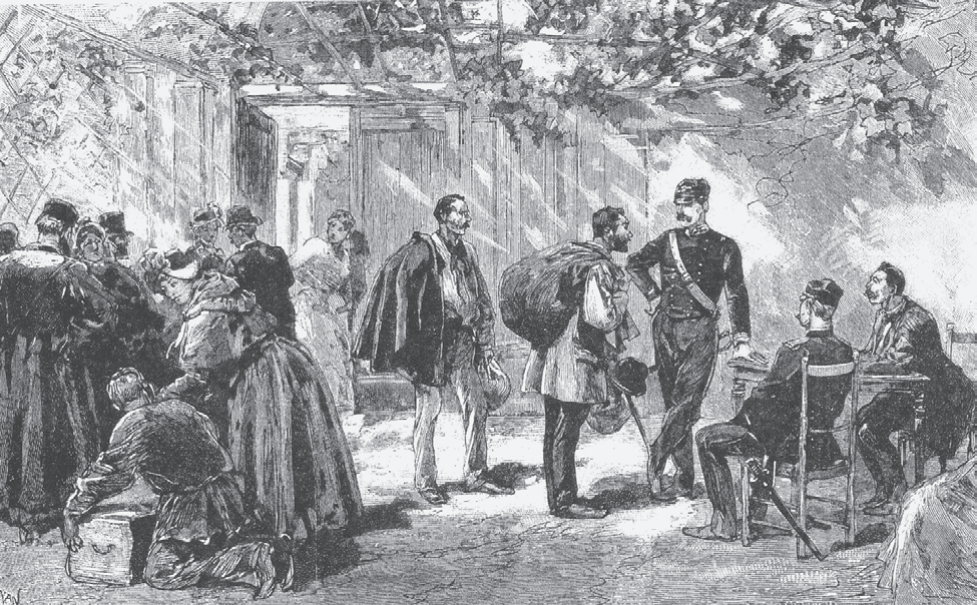
The control of travelers from cholera-affected countries, who were arriving by land at the France–Italy border during the cholera epidemic of 1865–1866. (Photograph in the author's possession).

Anticontagionists, who disbelieved the communicability of cholera, contested quarantine and alleged that the practice was a relic of the past, useless, and damaging to commerce. They complained that the free movement of travelers was hindered by sanitary cordons and by controls at border crossings, which included fumigation and disinfection of clothes (Figures 1,2,3). In addition, quarantine inspired a false sense of security, which was dangerous to public health because it diverted persons from taking the correct precautions. International cooperation and coordination was stymied by the lack of agreement regarding the use of quarantine. The discussion among scientists, health administrators, diplomatic bureaucracies, and governments dragged on for decades, as demonstrated in the debates in the International Sanitary Conferences (*31*), particularly after the opening, in 1869, of the Suez Canal, which was perceived as a gate for the diseases of the Orient (*32*). Despite pervasive doubts regarding the effectiveness of quarantine, local authorities were reluctant to abandon the protection of the traditional strategies that provided an antidote to population panic, which, during a serious epidemic, could produce chaos and disrupt public order (*33*).

A turning point in the history of quarantine came after the pathogenic agents of the most feared epidemic diseases were identified between the nineteenth and twentieth centuries. International prophylaxis against cholera, plague, and yellow fever began to be considered separately. In light of the newer knowledge, a restructuring of the international regulations was approved in 1903 by the 11th Sanitary Conference, at which the famed convention of 184 articles was signed (*31*).

## Influenza

In 1911, the eleventh edition of Encyclopedia Britannica emphasized that “the old sanitary preventive system of detention of ships and men” was “a thing of the past” (*34*). At the time, the battle against infectious diseases seemed about to be won, and the old health practices would only be remembered as an archaic scientific fallacy. No one expected that within a few years, nations would again be forced to implement emergency measures in response to a tremendous health challenge, the 1918 influenza pandemic, which struck the world in 3 waves during 1918–1919 (Technical Appendix). At the time, the etiology of the disease was unknown. Most scientists thought that the pathogenic agent was a bacterium, *Haemophilus influenzae*, identified in 1892 by German bacteriologist Richard Pfeiffer (*35*).

During 1918–1919, in a world divided by war, the multilateral health surveillance systems, which had been laboriously built during the previous decades in Europe and the United States, were not helpful in controlling the influenza pandemic. The ancestor of the World Health Organization, the Office International d’Hygiène Publique, located in Paris (*31*), could not play any role during the outbreak. At the beginning of the pandemic, the medical officers of the army isolated soldiers with signs or symptoms, but the disease, which was extremely contagious, quickly spread, infecting persons in nearly every country. Various responses to the pandemic were tried. Health authorities in major cities of the Western world implemented a range of disease-containment strategies, including the closure of schools, churches, and theaters and the suspension of public gatherings. In Paris, a sporting event, in which 10,000 youths were to participate, was postponed (*36*). Yale University canceled all on-campus public meetings, and some churches in Italy suspended confessions and funeral ceremonies. Physicians encouraged the use of measures like respiratory hygiene and social distancing. However, the measures were implemented too late and in an uncoordinated manner, especially in war-torn areas where interventions (e.g., travel restrictions, border controls) were impractical, during a time when the movement of troops was facilitating the spread of the virus. 

In Italy, which along with Portugal had the highest mortality rate in Europe, schools were closed after the first case of the unusually severe hemorrhagic pneumonia; however, the decision to close schools was not simultaneously accepted by health and scholastic authorities (*37*). Decisions made by health authorities often seemed focused more on reassuring the public about efforts being made to stop transmission of the virus rather than on actually stopping transmission of the virus (*35*). Measures adopted in many countries disproportionately affected ethnic and marginalized groups. In colonial possessions (e.g., New Caledonia), restrictions on travel affected the local populations (*3*). The role that the media would play in influencing public opinion in the future began to take shape. Newspapers took conflicting positions on health measures and contributed to the spread of panic. The largest and most influential newspaper in Italy, Corriere della Sera, was forced by civil authorities to stop reporting the number of deaths (150–180 deaths/day) in Milan because the reports caused great anxiety among the citizenry. In war-torn nations, censorship caused a lack of communication and transparency regarding the decision-making process, leading to confusion and misunderstanding of disease-control measures and devices, such as face masks (ironically named “muzzles” in Italian) (*35*).

During the second influenza pandemic of the twentieth century, the “Asian flu” pandemic of 1957–1958, some countries implemented measures to control spread of the disease. The illness was generally milder than that caused by the 1918 influenza, and the global situation differed. Understanding of influenza had advanced greatly: the pathogenic agent had been identified in 1933, vaccines for seasonal epidemics were available, and antimicrobial drugs were available to treat complications. In addition, the World Health Organization had implemented a global influenza surveillance network that provided early warning when novel influenza (H2N2) virus, began spreading in China in February 1957 and worldwide later that year. Vaccines had been developed in Western countries but were not yet available when the pandemic began to spread simultaneously with the opening of schools in several countries. Control measures (e.g., closure of asylums and nurseries, bans on public gatherings) varied from country to country but, at best, merely postponed the onset of disease for a few weeks (*38*). This scenario was repeated during the influenza A(H3N2) pandemic of 1968–1969, the third and mildest influenza pandemic of the twentieth century. The virus was first detected in Hong Kong in early 1968 and was introduced into the United States in September 1968 by US Marines returning from Vietnam. In the winter of 1968–69, the virus spread around the world; the effect was limited and there were no specific containment measures.

A new chapter in the history of quarantine opened in the early twenty-first century as traditional intervention measures were resurrected in response to the global crisis precipitated by the emergence of SARS, an especially challenging threat to public health worldwide. SARS, which originated in Guangdong Province, China, in 2003, spread along air-travel routes and quickly became a global threat because of its rapid transmission and high mortality rate and because protective immunity in the general population, effective antiviral drugs, and vaccines were lacking. However, compared with influenza, SARS had lower infectivity and a longer incubation period, providing time for instituting a series of containment measures that worked well (*39*). The strategies varied among the countries hardest hit by SARS (People’s Republic of China and Hong Kong Special Administrative Region; Singapore; and Canada). In Canada, public health authorities asked persons who might have been exposed to SARS to voluntarily quarantine themselves. In China, police cordoned off buildings, organized checkpoints on roads, and even installed Web cameras in private homes. There was stronger control of persons in the lower social strata (village-level governments were empowered to isolate workers from SARS-affected areas). Public health officials in some areas resorted to repressive police measures, using laws with extremely severe punishments (including the death penalty), against those who violated quarantine. As had occurred in the past, the strategies adopted in some countries during this public health emergency contributed to the discrimination and stigmatization of persons and communities and raised protests and complaints against limitations and travel restrictions.

## Conclusions

More than half a millennium since quarantine became the core of a multicomponent strategy for controlling communicable disease outbreaks, traditional public health tools are being adapted to the nature of individual diseases and to the degree of risk for transmission and are being effectively used to contain outbreaks, such as the 2003 SARS outbreak and the 2009 influenza A(H1N1)pdm09 pandemic. The history of quarantine—how it began, how it was used in the past, and how it is used in the modern era—is a fascinating topic in history of sanitation. Over the centuries, from the time of the Black Death to the first pandemics of the twenty-first century, public health control measures have been an essential way to reduce contact between persons sick with a disease and persons susceptible to the disease. In the absence of pharmaceutical interventions, such measures helped contain infection, delay the spread of disease, avert terror and death, and maintain the infrastructure of society. 

Quarantine and other public health practices are effective and valuable ways to control communicable disease outbreaks and public anxiety, but these strategies have always been much debated, perceived as intrusive, and accompanied in every age and under all political regimes by an undercurrent of suspicion, distrust, and riots. These strategic measures have raised (and continue to raise) a variety of political, economic, social, and ethical issues (*39*,*40*). In the face of a dramatic health crisis, individual rights have often been trampled in the name of public good. The use of segregation or isolation to separate persons suspected of being infected has frequently violated the liberty of outwardly healthy persons, most often from lower classes, and ethnic and marginalized minority groups have been stigmatized and have faced discrimination. This feature, almost inherent in quarantine, traces a line of continuity from the time of plague to the 2009 influenza A(H1N1)pdm09 pandemic.

The historical perspective helps with understanding the extent to which panic, connected with social stigma and prejudice, frustrated public health efforts to control the spread of disease. During outbreaks of plague and cholera, the fear of discrimination and mandatory quarantine and isolation led the weakest social groups and minorities to escape affected areas and, thus, contribute to spreading the disease farther and faster, as occurred regularly in towns affected by deadly disease outbreaks. But in the globalized world, fear, alarm, and panic, augmented by global media, can spread farther and faster and, thus, play a larger role than in the past. Furthermore, in this setting, entire populations or segments of populations, not just persons or minority groups, are at risk of being stigmatized. In the face of new challenges posed in the twenty-first century by the increasing risk for the emergence and rapid spread of infectious diseases, quarantine and other public health tools remain central to public health preparedness. But these measures, by their nature, require vigilant attention to avoid causing prejudice and intolerance. Public trust must be gained through regular, transparent, and comprehensive communications that balance the risks and benefits of public health interventions. Successful responses to public health emergencies must heed the valuable lessons of the past (*39**,**40*).

Technical Appendix List of publications chronicling the influenza pandemic of 1918–1919.
