# Supplementary material for: Lessons from the History of Quarantine, from Plague to Influenza A
Source: Emerg Infect Dis. 2013 Feb;19(2):254–9. doi: 10.3201/eid1902.120312 (PMC3559034; doi:10.3201/eid1902.120312)
Supplement: Technical Appendix — List of publications chronicling the influenza pandemic of 1918–1919. [file 12-0312-Techapp-s1.pdf]

Article DOI: http://dx.doi.org/10.3201/eid1902.120312

Lessons from the History of Quarantine, from Plague to Influenza A

Technical Appendix

Since the end of the twentieth century, the worldwide influenza outbreak of 1918–1919 has been a focus of interest of researchers from different scientific backgrounds. More recently, the results of investigations on genetic features of the deadly 1918 influenza virus have aroused a new historical curiosity. Little by little, the general works that chronicled the pandemic have been augmented by studies on outbreaks in additional countries and cities or have focused on particular aspects of the pandemic, such as demographic factors. The following list, while by no means exhaustive, includes most of the key historical and contemporary literature on the subject:

Ammon CE. Spanish flu epidemic in 1918 in Geneva. Switzerland. Euro Surveill. 2002;7:190–2 . PubMed

Beveridge WIB. Influenza. The Last Great Plague London. London: Heinemann; 1977.

Chowell G, Bettencourt LM, Johnson N, Alonso WJ, Viboud C. The 1918–1919 influenza pandemic in England and Wales: spatial patterns in transmissibility and mortality impact. Proc Biol Sci. 2008;275:501–9. PubMed http://dx.doi.org/10.1098/rspb.2007.1477

Chowell G, Viboud C, Simonsen L, Miller MA, Acuna-Soto R. Mortality patterns associated with the 1918 influenza pandemic in Mexico: evidence for a spring herald wave and lack of preexisting immunity in older populations. J Infect Dis. 2010;202:567–75. PubMed http://dx.doi.org/10.1086/654897

Chowell G, Viboud C, Simonsen L, Miller MA, Hurtado J, Soto G. The 1918–1920 influenza pandemic in Peru. Vaccine. 2011;29(Suppl 2):B21–6. PubMed http://dx.doi.org/10.1016/j.vaccine.2011.02.048

Collier R. The Plague of the Spanish Lady. New York, NY: Atheneum; 1974.

Collins SD. Influenza in the United States, 1887–1956. Review and study of illness and medical care with special reference to long-time trends. Public Health Monograph No. 48. Washington (DC): US Department of Health, Education, and Welfare. Public Health Service; 1957.

Crosby A. America’s forgotten pandemic: the influenza of 1918. New York: Cambridge University Press; 1989.

Dávila B. La gripe española. La pandemia de 1918-1919. Madrid: Siglo XXI de España y Centro de Investigaciones Sociológicas 1993.

Erkoreka A. The Spanish influenza pandemic in occidental Europe (1918–1920) and victim age. Influenza Other Respi Viruses. 2010;4:81–9. PubMed http://dx.doi.org/10.1111/j.1750-2659.2009.00125.x

Ghendon Y. Introduction to pandemic influenza through history. Eur J Epidemiol. 1994;10:451–3. PubMed http://dx.doi.org/10.1007/BF01719673

Iezzoni I. Influenza 1918: the worst epidemic in American history. New York: TV Books; 1999.

Johnson NP, Mueller J. Updating the accounts: global mortality of the 1918–1920 “Spanish” influenza pandemic. Bull Hist Med. 2002;76:105–15. PubMed http://dx.doi.org/10.1353/bhm.2002.0022

Jordan. E.O. Epidemic Influenza, a Survey, Chicago : American Medical Association; 1927.

Killingray D. The influenza pandemic of 1918–1919 in the British Caribbean. Soc Hist Med. 1994;7:59–87. PubMed http://dx.doi.org/10.1093/shm/7.1.59

Kolata G. Flu: The story of the great influenza pandemic of 1918 and the search for the virus that caused it. New York: Farrar, Straus, & Giroux; 1999.

Langford C. The Age Pattern of Mortality in the 1918-19 Influenza Pandemic: An Attempted Explanation based on Data for England and Wales. Med Hist. 2002;46:1–20 . PubMed

Lee VJ, Chen MI, Chan SP, Wong CS, Cutter J, Goh KT. Influenza pandemics in Singapore, a tropical, globally connected city. Emerg Infect Dis. 2007;13:1052–7. PubMed http://dx.doi.org/10.3201/eid1307.061313

Mills ID. The 1918-19 influenza pandemic: the Indian experience. Indian Econ Soc Hist Rev. 1986;23:1–40. PubMed http://dx.doi.org/10.1177/001946468602300102

Morens DM, Taubenberger JK, Harvey HA, Memoli MJ. The 1918 influenza pandemic: lessons for 2009 and the future. Crit Care Med. 2010;38(Suppl):e10–20. PubMed http://dx.doi.org/10.1097/CCM.0b013e3181ceb25b

Olson DR, Simonsen L, Edelson PJ, Morse SS. Epidemiological evidence of an early wave of the 1918 influenza pandemic in New York City. Proc Natl Acad Sci U S A. 2005;102:11059–63. PubMed http://dx.doi.org/10.1073/pnas.0408290102

Patterson KD, Pyle GF. The geography and mortality of the 1918 influenza pandemic. Bull Hist Med. 1991;65:4–21 . PubMed

Patterson KD, Pyle GF. The diffusion of influenza in sub-Saharan Africa during the 1918–1919 pandemic. Soc Sci Med. 1983;17:1299–307. PubMed http://dx.doi.org/10.1016/0277-9536(83)90022-9

Phillips H. Black October’: the impact of the Spanish influenza epidemic of 1918 in South Africa, Pretoria: The Government Printer; 1990.

Potter CW. Chronicle of influenza pandemics' in Nicholson KG, Webster RG, Hay. AJ, eds. Textbook of influenza. London: Blackwell Scientific. Publications; 1998.

Richard SA, Sugaya N, Simonsen L, Miller MA, Viboud C. A comparative study of the 1918–1920 influenza pandemic in Japan, USA and UK: mortality impact and implications for pandemic planning. Epidemiol Infect. 2009;137:1062–72. PubMed http://dx.doi.org/10.1017/S0950268809002088

Stern AM, Cetron MS, Markel H. Closing the schools: lessons from the 1918-19 U.S. influenza pandemic. Health Aff (Millwood). 2009;28:w1066–78. PubMed http://dx.doi.org/10.1377/hlthaff.28.6.w1066

Stern AM, Cetron MS, Markel H. The 1918-1919 influenza pandemic in the United States: lessons learned and challenges exposed. Public Health Rep. 2010;125(Suppl 3):6–8 . PubMed

Taubenberger JK, Morens DM. 1918 Influenza: the mother of all pandemics. Emerg Infect Dis. 2006;12:15–22 . PubMed

Tognotti E. La Spagnola in Italia. Storia dell'influenza che fece temere la fine del mondo (1918–19). Milano: Franco Angeli; 2002.
